# Disruption of the Serine/Threonine Kinase *Akt* Gene Affects Ovarian Development and Fecundity in the Cigarette Beetle, *Lasioderma serricorne*

**DOI:** 10.3389/fphys.2021.765819

**Published:** 2021-10-07

**Authors:** Kang-Kang Xu, Yi Yan, Shu-Yan Yan, Peng-Liang Xia, Wen-Jia Yang, Can Li, Hong Yang

**Affiliations:** ^1^Guizhou Provincial Key Laboratory for Agricultural Pest Management of Mountainous Region, Institute of Entomology, Guizhou University, Guiyang, China; ^2^Guizhou Provincial Key Laboratory for Rare Animal and Economic Insect of the Mountainous Region, College of Biology and Environmental Engineering, Guiyang University, Guiyang, China; ^3^Enshi Tobacco Company of Hubei Province, Enshi, China; ^4^College of Tobacco Science, Guizhou University, Guiyang, China

**Keywords:** serine/threonine kinase, cigarette beetle, insulin, reproduction, juvenile hormone, carbohydrate metabolism

## Abstract

Serine/threonine kinase Akt, an important component of the insulin signaling pathway, plays an essential role in many physiological processes. In this study, we identified and characterized an *Akt* gene (designated *LsAkt*) from the cigarette beetle, *Lasioderma serricorne*. *LsAkt* contains a 1614 bp open reading frame encoding a 537 amino acid protein that possesses a conserved pleckstrin homology domain and a serine/threonine kinase domain. The expression of *LsAkt* was high in pupal stages and peaked in day-4 female pupae. In adult tissues, *LsAkt* was highly expressed in the thorax, ovary, and midgut. The expression of *LsAkt* was induced by methoprene or bovine insulin *in vivo*, but significantly decreased by 20-hydroxyecdysone. RNA interference-mediated knockdown of *LsAkt* resulted in severely blocked ovarian development and reduced fecundity and hatchability. The vitellogenin (Vg) content and juvenile hormone (JH) titers of *LsAkt*-depletion beetles were decreased, and expressions of *Vg* and four JH signaling and biosynthetic genes were significantly decreased. Silencing of *LsAkt* reduced the amounts of glucose, glycogen, and trehalose in female adults and affected the expressions of seven key carbohydrate metabolic genes. Taken together, it is inferred that *Akt* implicates in *L. serricorne* reproduction by modification of Vg synthesis, juvenile hormone production and carbohydrate metabolism.

## Introduction

Insulins are multifunctional peptide hormones and consist of insulin-like peptide (ILP), insulin-like growth factor (IGF), and relaxin (Wu and Brown, 2006). Insulin structure, function, and signal transduction are evolutionary conserved in both vertebrates and invertebrates (Taniguchi et al., 2006; Das and Dobens, 2015). In insects, ILPs specifically activate the insulin receptors, which in turn, transmit a signal via the phosphoinostide 3-kinase (PI3K)-serine/threonine kinase (Akt) pathway or the mitogen-activated protein kinase (MAPK) pathway. This mediates diverse physiological events including growth, metabolism, longevity, and reproduction (Zhang et al., 2009; Das and Arur, 2017; Xu et al., 2021).

Serine/threonine kinase Akt (also known as protein kinase B, PKB), transduces the insulin signal through the phosphorylation of several downstream proteins such as other kinases, signaling proteins, and transcription factors (Verdu et al., 1999; Teleman, 2010). In insects, Akt helps regulate development, behavior, reproduction, lifespan, and stress resistance. For example, Akt functions in the regulation of apoptosis and cell size during *Drosophila* development (Staveley et al., 1998; Scanga et al., 2000). In *Haemaphysalis longicornis*, RNA interference (RNAi)-mediated knockdown of *HlAkt* inhibited blood feeding and arrested internal organ growth (Umemiya-Shirafuji et al., 2012). Akt phophorylation is associated with the embryonic diapause process of *Bombyx mori* (Gu et al., 2019). In *Maruca vitrata*, silencing of *Akt* significantly decreased larval growth rate and pupal weight (Al Baki et al., 2019). Bioassay analysis by sparing with a mixture of transformed *Escherichia coli* expressing ds*Akt* and *Bacillus thuringiensis* (Bt) in fourth-instar larvae of *M. vitrata* produced higher control mortality than a single treatment with Bt alone or ds*Akt* alone (Al Baki et al., 2020). In *Chrysopa pallens*, knockdown of *Akt* suppressed the expression of the *vitellogenin* (*Vg*) gene, hampered ovarian development, and reduced egg mass and hatching rate (Han et al., 2020). By contrast, over-expression of active *Akt* in the fat body of *Anopheles stephensi* extended the lifespan and increased fecundity of females (Hun et al., 2019). In *Aedes aegypti*, depletion of *Akt* significantly decreased the 4E-binding protein (4E-BP) phosphorylation and reduced the lifespan of adult females (Roy and Raikhel, 2012). Increased *Akt* signaling in the midguts of *A. stephensi* females significantly reduced their longevity (Arik et al., 2015). Akt signal transduction also is involved in the cold hardiness of *Epiblema scudderiana* larvae (Zhang and Storey, 2017).

Juvenile hormone (JH) and 20-hydroxyecdysone (20E), as well as insulin-like peptides, are components of an endocrine network in insects that coordinate to regulate development, molting, and reproduction. For example, insulin signaling regulates *Drosophila* larval molting by controlling the synthesis of ecdysone in the prothoracic glands (Walkiewicz and Stern, 2009). In *Tribolium castaneum*, JH functions in the regulation of Vg synthesis in the fat body via an insulin signaling cascade (Sheng et al., 2011). In the prothoracic glands of *B. mori*, bovine insulin can enhance the phosphorylation of Akt and stimulate ecdysteriod secretion (Gu et al., 2011). In *Manduca sexta*, Akt phosphorylation level stimulation by insulin was a non-requisite step in ecdysone secretion (Smith et al., 2014). However, the interaction among the JH, 20E, and insulin signaling remains poorly understood.

The cigarette beetle, *Lasioderma serricorne* (Coleoptera: Anobiidae), is a destructive stored pest in the tobacco and food industry and occurs worldwide (Ashworth, 1993). *L. serricorne* larvae cause economic damage to stored materials by direct damage and production of fecal material (Riudavets et al., 2007). This species has high reproductive potential. A single female beetle can lay 10–100 eggs during its 9–11 d oviposition period (Sivik et al., 1957). Previous studies have focused on the biology and ecology of *L. serricorne* and its control (Edde, 2019; Yang et al., 2020). However, knowledge of the reproductive physiology of *L. serricorne* remains limited, and the molecular mechanism of *L. serricorne* reproduction is unknown. Here we report (1) the full-length open reading frame (ORF) sequence of *Akt* (*LsAkt*) in *L. serricorne*, (2) the expression profiles of *LsAkt* in different developmental stages and tissues, as well as in response to exogenous hormone treatments, (3) functional analysis of *LsAkt* by RNAi in ovarian development and female fecundity, and (4) the effects of *LsAkt* RNAi on Vg synthesis, JH production, and carbohydrate metabolism.

## Materials and Methods

### Insects

The *L. serricorne* strain was originally collected in 2014 from a tobacco warehouse in Guizhou province, China. Stock colonies were maintained in the laboratory at 28°C with a relative humidity of 40% and constant (24 h) darkness. The rearing method was described in a previous report (Chen et al., 2018). Under laboratory-reared conditions, they stay at the pupal stage for 5 days, and the ovaries of female adults mature 5 days post eclosion.

### Gene Cloning and Sequence Analysis

Total RNA was extracted from *L. serricorne* adults using a MiniBEST Universal Extraction Kit (TaKaRa, Dalian, China). One microgram of RNA was used to synthesize the first-strand cDNA by TransScript Synthesis Supermix (TransGen Biotech, Beijing, China). One unigene cDNA encoding serine/threonine kinase Akt was obtained from a *L. serricorne* transcriptomic database (SRR13065789). The full-length cDNA sequence of *LsAkt* was verified by reverse transcription PCR using gene-specific primers (Supplementary Table 1). Amplified PCR product was inserted into *pEASY*^®^ –T1 vector (TransGen Biotech) and then sequenced (Tsingke Bio, Chengdu, China).

The nucleotide sequence similarities were identified by using the National Center for Biotechnology Information (NCBI) basic local alignment search tool.^1^ The coding sequence was predicted by NCBI ORF Finder,^2^ and the putative amino acid sequences were deduced by using DNAMAN7 (Lynnon Biosoft, Vaudreuil, Quebec, Canada). Molecular weight and isoelectric point were determined using ExPASy Proteomics Server.^3^ Conserved domains were determined by using the Simple Modular Architecture Research Tool.^4^ Multiple amino acid sequence alignment was performed by Clustal X (Larkin et al., 2007). A neighbor-joining phylogenetic tree was constructed using MEGA7 (Kumar et al., 2016) with 1000 bootstrap replications, and evolutionary relationship of *LsAkt* was determined based on insect Akt sequences available in the NCBI GenBank database (Supplementary Table 2).

### Spatio-Temporal Expression Pattern of *LsAkt*

For the temporal expression profile of *LsAkt*, whole bodies of *L. serricorne* at various developmental stages, including pupae (1–5 d old) and adults (1–5 d old) were collected. For tissue-specific expression analysis, seven samples dissected from day-5 adult females were prepared, including head, thorax, epidermis, midgut, fat body, and ovary. Each sample included 10–50 individuals, and three biological replicates were performed. Total RNA extraction and cDNA synthesis were performed as described above. The mRNA levels of *LsAkt* were determined by Quantitative real-time PCR (qPCR) using TransStart^®^ Top Green qPCR SuperMix (TransGen) with the CFX-96 real-time PCR system (Bio-Rad, Hercules, CA, United States). The reaction conditions were as follows: denaturation for 3 min at 94°C, followed by 40 cycles at 94°C for 5 s and 60°C for 30 s. A melting curve was used to further assess the qPCR primer specificity. Relative mRNA levels of target genes were normalized by the stable reference genes *elongation factor 1-alpha* (*EF1*α) and *18S ribosomal RNA* (*18S*) using the 2^−ΔΔ*Ct*^ method (Livak and Schmittgen, 2001; Yang et al., 2020).

### Hormone Treatment

To examine the effect of exogenous hormones on *LsAkt* expression in pupae, the JH analog methoprene (purity: 95%) (Sigma-Aldrich, St. Louis, MO, United States), 20E (HPLC: ≥ 95% purity), and bovine insulin (purity: ≥ 27 USP units/mg) (Sigma-Aldrich) were used *in vivo*. In brief, a 10 μg/μL stock solution of methoprene or 20E dissolved in 95% ethanol were diluted to 1.0 mg/mL with distilled water. Either methoprene (200 ng/pupa) or 20E solution (120 ng/pupa) was then injected into day-3 pupae using a Nanoliter 2010 injector (World Precision Instruments, Sarasota, FL, United States). Pupae treated with an equivalent amount of ethanol were used as a control. For the insulin treatment, bovine insulin was solubilized in 25 mM HEPES (pH 8.2) and then diluted with distilled water to a final concentration of 3.0 mg/mL. Each pupa was injected with 100 nL insulin solution (300 ng/pupa), and controls were treated with an equal volume of HEPES buffer. Thirty individuals were randomly selected from each group at 3, 6, 12, and 24 h after injection, and the expression of *LsAkt* was determined by qPCR. Three biological replicates were used for each treatment.

### RNAi and Fecundity Assay

RNA interference (RNAi) was performed to investigate the potential function of *LsAkt* in *L. serricorne*. The dsRNAs against *LsAkt* and *green fluorescent protein* (*GFP*, as control) were synthesized using a TranscriptAid T7 High Yield Transcription Kit (Thermo Scientific, Wilmington, DE, United States). The dsRNA was purified by phenol/chloroform solution, precipitated by ethanol, and dissolved in nuclease-free water. Day-3 female pupae were used in the RNAi experiments. Approximately 0.2 μL dsRNAs of *LsAkt* or *GFP* (1.5 μg/μL) were injected into each individual. All the dsRNA-treated insects were reared under the conditions stated above. At 72, 96, and 120 h after injection, female individuals (*n* = 20 each of three replicates) were collected to examine the efficiency of RNAi by qPCR. Day-3 female pupae were challenged with dsRNAs targeting *LsAkt* or *GFP* and emerged into adults. New emerged females at 0–6 h eclosion from the dsRNA injected pupae were collected for the paired mating assay. Each dsRNA-treated female was paired with one non-treated same age male in a petri dish for mating. Insects treated with ds*GFP* served as a parallel control. Each of the adult pairs was transferred daily into a new Petri dish containing artificial diet. The number of eggs laid was recorded each day using a stereomicroscope (Olympus SZX12, Tokyo, Japan). The hatching rate was computed every 12 h for 5 d until the unhatched eggs started to shrink. Thirty pairs of *L. serricorne* were used to analyze fecundity and hatchability for each treatment. To verify the effects of *LsAkt* on the oogenesis and ovary development of *L. serricorne*, the day-5 female adults from each treatment group were dissected. The ovaries were dissected in 1 × phosphate buffer saline (PBS) and photographed with a stereomicroscope VHX-6000 (Keyence Corporation, Osaka, Japan). The lengths of ovarian tube and lateral oviduct were measured using the Keyence application suite software.

### Effect of *LsAkt* on Vitellogenin Content, Juvenile Hormone Titers, and Expression of Reproductive Genes

To study the effects of *LsAkt* RNAi on reproduction, samples were collected from pupae injected with ds*LsAkt* and ds*GFP* for 5 d. About 20 individuals were pooled as one sample, and three replications were performed. Each sample was weighed and then homogenized with a corresponding volume of PBS at the ratio of 1 g: 9 mL and centrifuged at 2500 g, 4°C for 20 min. The supernatants were collected for measurement of vitellogenin content according to the instructions of the Insect Vitellogenin Enzyme-linked immunosorbent assay (ELISA) Kit (Shanghai Meilian Biotechnology Co., Ltd). For JH titer determination, the supernatants were colleted for measurement of JH titers using the Insect JH ELISA Kit (Shanghai Meilian Biotechnology Co., Ltd). The relative expression profiles of *Vg*, *VgR*, and ten JH signaling and metabolic genes (Supplementary Table 1), were determined by qPCR as noted above. Thirty insects were treated as one replication, and three replications were performed.

### Effect of *LsAkt* on Carbohydrate Metabolism

To explore the effects of *LsAkt* RNAi on carbohydrate metabolism, the glucose, glycogen, and trehalose content assay was performed using a previously reported method (Xu et al., 2020). The contents of glucose, glycogen, and trehalose in whole insect bodies were measured by the SpectraMax M2 microplate reader (Molecular Devices, Sunnyvale, CA, United States) at 5 d after dsRNA treatment. Each sample contained 50 individuals, and three biological replications were prepared. The insect samples were homogenized in 0.25 M Na_2_CO_3_ and then incubated at 70°C for 10 min. By adding 0.2 M Na-acetate and 1 M acetic acid, the mixture was adjusted to pH 5.2. To measure the glycogen content, one half of the mixture was incubated with amyloglucosidase (Sigma-Aldrich). For trehalose measurement, the other half of the mixture was incubated with trehalase (Sigma-Aldrich) at 37°C, and the treated insects were homogenized in PBS (pH 7.4, 137 mM NaCl, 10 mM Na2HPO4, 2.7 mM KCl, and 2 mM KH_2_PO_4__)_. The glucose level was determined using a glucose (GO) assay kit (Sigma-Aldrich) according to the manufacturer’s instructions. After *LsAkt* was knocked down in *L. serricorne*, the transcript levels of eleven carbohydrate metabolic genes (Supplementary Table 1) were detected by qPCR at 5 d after injection.

### Statistical Analysis

Statistical analyses were performed using GraphPad Prism version 6.01 software (GraphPad software, La Jolla, CA, United States). Significant differences between two samples and among multiple samples were determined by one-tailed Student’s *t*-test and a one-way analysis of variance followed by a least significant difference test, respectively.

## Results

### Identification and Sequence Analysis of *LsAkt*

**FIGURE 1:**
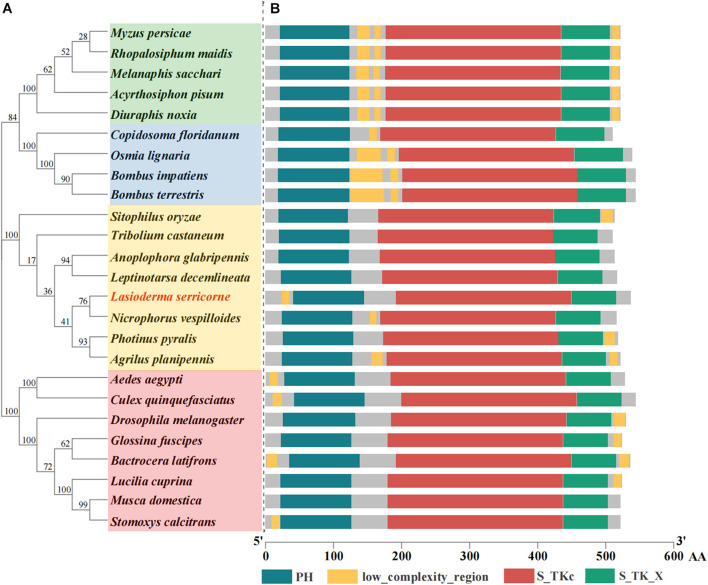
Phylogenetic analysis and protein structure comparison of insect Akts. **(A)** Phylogenetic tree generated with MEGA 7 by using neighbor-joining method with 1000 bootstrap replications. The GenBank accession numbers are listed in Supplementary Table 2. **(B)** Schematic alignment and comparison of domain architecture of insect Akts. AA, amino acids; PH, pleckstrin homology; S_TKc, catalytic domain; S_TK_X, the extension to serine/threonine-protein kinase.

The ORF of *LsAkt* is 1614 bp encoding a putative protein of 537 amino acids (GenBank accession number: MZ695806). The predicted molecular weight of *LsAkt* was 61.33 kDa and the isoelectric point was 5.98. Multiple sequence alignment revealed that LsAkt shares high conservation with other insects in the typical motifs of the Akts, harboring a pleckstrin homology (PH) domain, low complexity region, catalytic domain (S_TKc), and the extension to serine/threonine kinases (S_TK_X) (Figure 1). LsAkt protein contains putative phosphorylation sites, including 28, 26, and 8 sites for serine, threonine, and tyrosine, respectively (Supplementary Figure 1). *LsAkt* shared a high amino acid identity with other Coleoptera; 85.14% identity with *Nicrophorus vespilloides* (XP_017772201.1), 84.64% with *Leptinotarsa decemlineata* (XP_023017655.1), 84.14% with *Anoplophora glabripennis* (XP_018571651.1), and 81.43% with *T. castaneum* (XP_008191524.1). Phylogenetic analysis of Akt from different insect species showed that LsAkt has a close relationship to other Coleoptera and is clustered with *N. vespilloides* (Figure 1).

### Developmental and Tissue-Specific Expression of *LsAkt*

**FIGURE 2:**
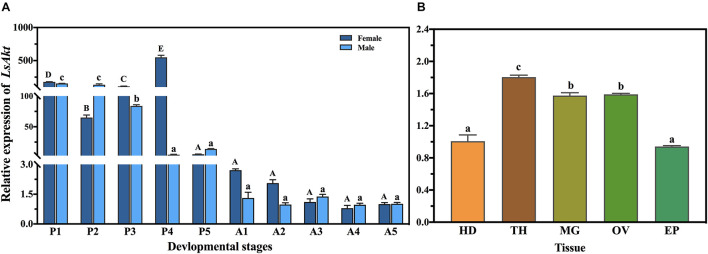
Developmental and tissue-specific expression patterns of *LsAkt*. **(A)** Temporal expression of *LsAkt* at different developmental stages. P1–P5, days 1–5 of the pupae; A1–5, days 1–5 of the adults. **(B)** Tissue distribution of *LsAkt* transcript in day-5 adults. HD, head; TH, thorax; MG, midgut; OV, ovary; EP, epidermis. Different uppercase letters above bars indicate significant differences of female insects, lowercase letters indicate significant differences of male insects or tissues based on one-way ANOVA followed by a least significant difference test (*P* < 0.05).

The qPCR was used to analyze the expression profiles of *LsAkt* on different developmental days and in different tissues. *LsAkt* was continuously expressed during the life stages tested. The expression of *LsAkt* remained at high levels in the pupal stages and had the highest expression levels in the day-4 female pupae. *LsAkt* had lower expression in the adult stages (Figure 2A). Tissue examination showed that *LsAkt* was expressed in all the selected tissues of day-5 adults, with relatively higher expression in the thorax, ovary, and midgut (Figure 2B).

### Effect of Exogenous Hormones on *LsAkt* Expression

**FIGURE 3:**
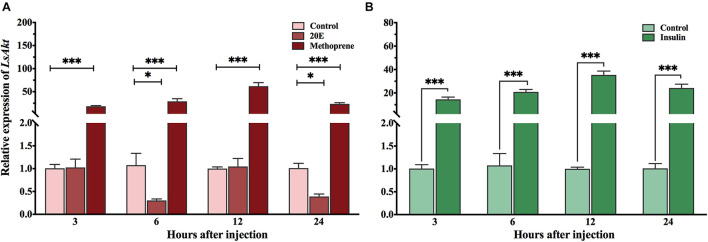
Expression profiles of *LsAkt* after exogenous hormone treatments. **(A)** Effect of 20E and methoprene on the expression of *LsAkt*. Control: insects injected with distilled water containing 0.1% ethanol; 20E: insects injected with 20E (120 ng/pupa); Methoprene; insects injected with methoprene (200 ng/pupa). **(B)** Effect of insulin on the expression of *LsAkt*. Control: insects injected with HEPES buffer; insulin, insects injected with bovine insulin (300 ng/pupa). Significant differences between treatment group and control group at the same point were determined using Student’s *t*-test (^∗^ *P* < 0.05, ^∗∗∗^ *P* < 0.001).

To test whether *LsAkt* could be induced by exogenous hormones, the expression profiles of *LsAkt* were determined by qPCR at the same time points. The expression of *LsAkt* was significantly upregulated by the JH analog methoprene compared with the control group with 18. 5-, 29. 1-, and 23.6-fold increase at 3, 6, and 24 h, respectively. The transcript level of *LsAkt* reached a peak (62.0-fold) at 12 h after methoprene exposure. Conversely, the expression of *LsAkt* was significantly downregulated at 6 and 24 h after 20E injection compared with the control (Figure 3A). The expression of *LsAkt* was significantly increased up to 14. 6-, 20. 9-, 35. 4-, and 24.2-fold at 3, 6, 12, and 24 h after insulin treatment compared with the control (Figure 3B).

### Knockdown of *LsAkt* Impairs Female Fecundity and Ovarian Development

**FIGURE 4:**
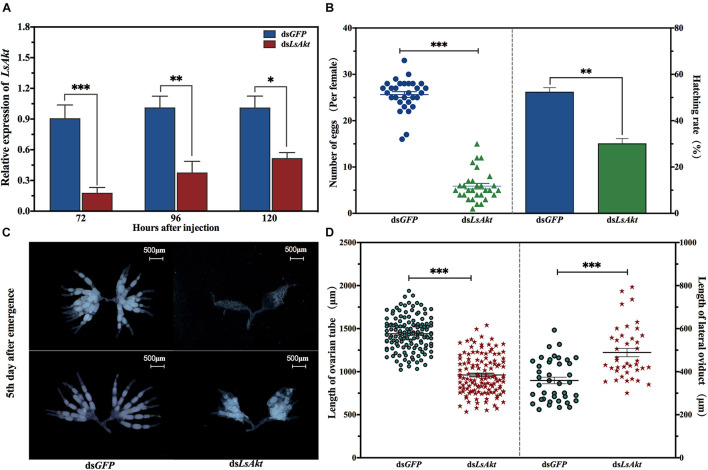
Effect of *LsAkt* RNAi on female reproduction in *L. serricorne*. **(A)** Relative expression levels of *LsAkt* at 72, 96, and 120 h after *LsAkt* or *GFP* dsRNA injection. **(B)** Determination of female fecundity after *LsAkt* knockdown. **(C)** Ovary morphology after *LsAkt* knockdown. Day-3 female pupae were injected with *LsAkt* or *GFP* dsRNA. Ovaries were dissected and photographed on the fifth day after adult eclosion. **(D)** Effects of *LsAkt* knockdown on the lengths of ovarian tube and lateral oviduct. Significant differences between the RNAi group and control group were determined using Student’s *t*-test (^∗^ *P* < 0.05, ^∗∗^ *P* < 0.01, ^∗∗∗^ *P* < 0.001).

To characterize the role of *LsAkt* in the reproductive process of *L. serricorne*, dsRNAs targeting *LsAkt* and *GFP* were injected into day-3 female pupae. Compared with the control, the expression level of *LsAkt* significantly decreased by 80.2%, 62.7%, and 48.7% at 72, 96, and 120 h, respectively, after ds*LsAkt* injection (Figure 4A). Notably, depletion of *LsAkt* had no negative effect on the pupa-adult transition. Female pupae were alive and successfully molted to adults at day 3 after injection with ds*LsAkt* or ds*GFP*. Newly emerged female adults were collected and allowed to pair with one wild-type male. Each ds*LsAkt*-treated female laid an average of 5.9 eggs, whereas the ds*GFP*-injected controls laid an average of 25.6 eggs per female. The egg hatchability in *LsAkt*-deficient females was significantly reduced by 42.5% compared with ds*GFP*-treated controls (*P* < 0.01) (Figure 4B).

Because *LsAkt* knockdown caused female reproductive deficiency, we examined ovarian development after dsRNA treatment. Females injected with ds*LsAkt* showed severely blocked in ovarian development. The abnormal ovaries had many non-vitellogenic oocytes and fewer mature eggs with less yolk protein deposition. In contrast, the ovaries of ds*GFP*-injected females were completely filled with regular banana-shaped oocytes, which were closely arranged in the ovarioles (Figure 4C). The lengths of ovarian tubes of the ds*LsAkt* group were significantly shorter than those of the ds*GFP* group, while the lengths of the lateral oviducts were longer than the controls (Figure 4D).

### Knockdown of *LsAkt* Disturbs Vitellogenin Synthesis and Juvenile Hormone Signal

**FIGURE 5:**
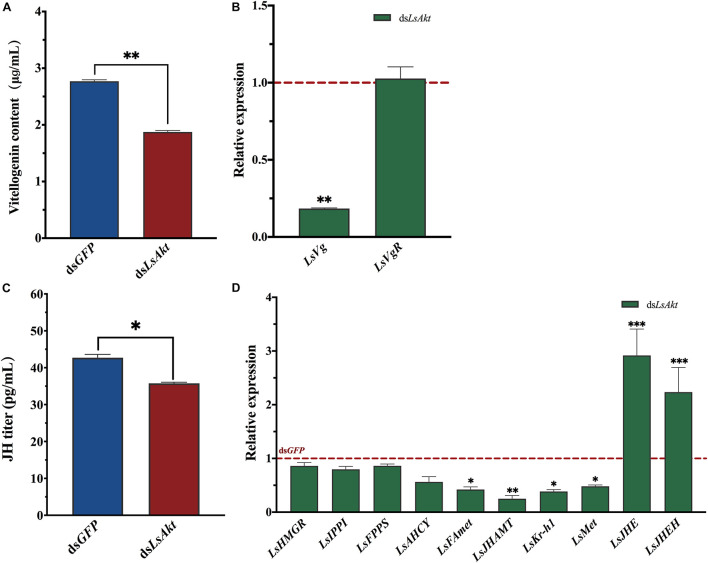
Effect of *LsAkt* RNAi on vitellogenin synthesis and juvenile hormone signaling. **(A)** Vitellogenin content in female adults after *LsAkt* knockdown. **(B)** Relative expression of *Vg* and *VgR* after RNAi. **(C)** JH tiers in female adults after *LsAkt* knockdown. **(D)** Relative expression of juvenile hormone signaling pathway genes after RNAi. The expression values were calculated by comparison to the ds*GFP* group, which was normalized at 1. Significant differences between the RNAi group and control group were determined using Student’s *t*-test (^∗^ *P* < 0.05, ^∗∗^ *P* < 0.01, ^∗∗∗^ *P* < 0.001).

The vitellogenin content was significantly decreased by 32.3% at 120 h after injection with ds*LsAkt* (*P* < 0.01) compared with the ds*GFP* group (Figure 5A). The expression of *LsVg* was significantly reduced by 81.8% in the *LsAkt*-depleted beetles. However, the expression of *LsVgR* did not vary significantly between the ds*LsAkt* and ds*GFP* females (Figure 5B). The JH titers of *LsAkt* knockdown individuals were significantly lower than those injected with ds*GFP* (*P* < 0.05) (Figure 5C). The mRNA levels of four JH synthesis and signaling genes, including *JH methyltransferase* (*LsJHAMT*), *farnesoic acid O-methyltransferase* (*LsFAmet*), *methoprene-tolerant* (*LsMET*), and *krüppel homolog 1* (*LsKr-h1*) were significantly decreased after knockdown of *LsAkt*. In contrast, the expressions of *JH esterase* (*LsJHE*) and *JH epoxide hydrolase* (*LsJHEH*) were increased (Figure 5D).

### Knockdown of *LsAkt* Affects Carbohydrate Metabolism

**FIGURE 6:**
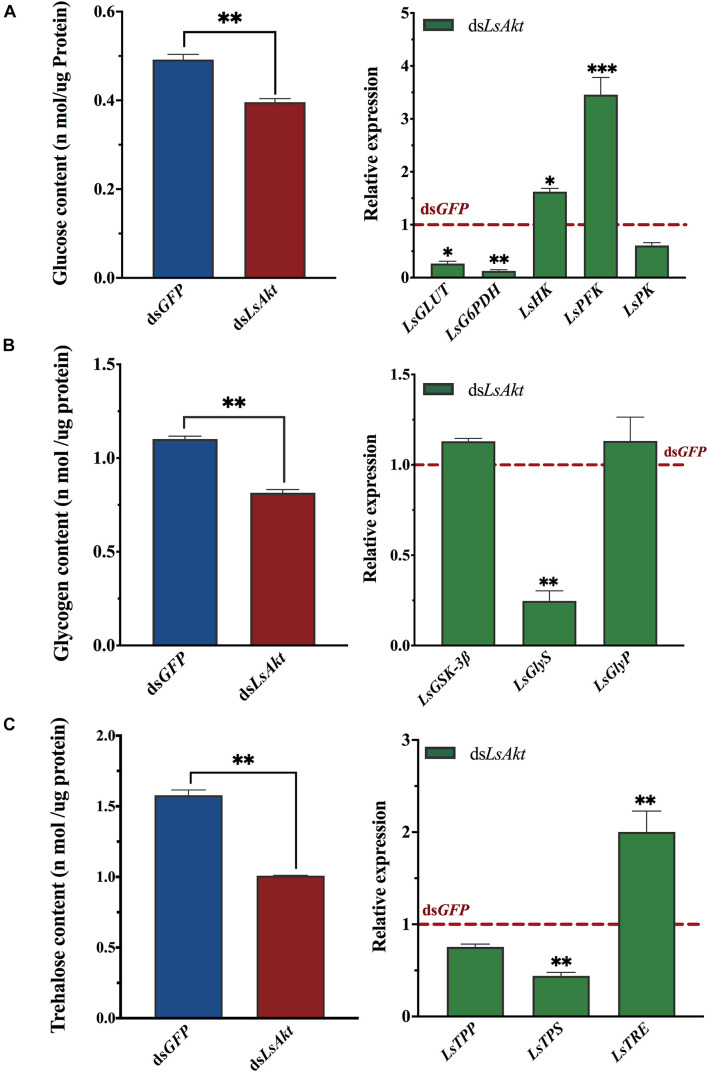
Effect of *LsAkt* RNAi on carbohydrate metabolism of *L. serricorne.* **(A)** Effects of *LsAkt* knockdown on glucose content and expression of four glucose-related genes. **(B)** Effects of *LsAkt* knockdown on glycogen content and expression of glycolytic pathway genes. **(C)** Effects of *LsAkt* knockdown on trehalose content and expression of three trehalose metabolic genes. The expression values were calculated by comparison to the ds*GFP* group, which was normalized at 1. Significant differences between the RNAi group and control group were determined using Student’s *t*-test (^∗^ *P* < 0.05, ^∗∗^ *P* < 0.01, ^∗∗∗^ *P* < 0.001).

We also used RNAi to investigate the roles of *LsAkt* in carbohydrate metabolism of *L. serricorne*. The contents of glucose, glycogen, and trehalose were significantly decreased in *LsAkt*-depleted beetles (*P* < 0.01). Also, the expression levels of *glucose transporter* (*LsGLUT*), *glucose-6-phosphate dehydrogenase* (*LsG6PDH*), *glycogen synthase* (*LsGlys*), and a *trehalose synthase* (*LsTPS*) were significantly decreased after knockdown of *LsAkt*. In contrast, the expression levels of *hexokinase* (*LsHK*), *phosphofructokinase* (*LsPFK*), and *trehalase* (*LsTRE*) were significantly upregulated compared with those in control insects (Figure 6).

## Discussion

Spatio-temporal expression analysis of *LsAkt* revealed ubiquitous expression in all developmental stages and tissues. The highest expression levels of *LsAkt* occurred in the pupal stage, which is the key preparation period for *L. serricorne* reproductive development. Expression of the *Akt* gene varies among insect species. In *A. aegypti*, *Akt* was only expressed in the early stages of embryos and in adult females (Riehle and Brown, 2003). Dramatically increased expression of *Akt* occurred in fifth-instar larvae and pre-pupae of *B. mori*, indicating that *Akt* may be related to metamorphosis in this species (Liu et al., 2010). Insect *Akt* expression also exhibits tissue specificity. In *B. dorsalis*, *Akt* was highly expressed in the midgut and Malpighian tubules (Xu K. K. et al., 2015). In *A. aegypti*, *Akt* was specifically expressed in the ovary of adult females (Riehle and Brown, 2003). *In situ hybridization* of *Diacamma sp*. ovaries revealed that *DiaAkt* was expressed in nurse cells, oocytes, and upper germarial regions of mated egglaying workers (Okada et al., 2010). Similar results were observed in this study showing that *LsAkt* was expressed primarily in the ovary of *L. serricorne*. However, there was non-negligible expression of *LsAkt* in other tissues, especially in the thorax and midgut, and its functions in these tissues are not known.

There is a functional relationship between hormones and the insulin signaling pathway. Dramatically increased expression of *Akt* by JH occurred in newly molted fourth instar larvae of *B. mori* (Cheng et al., 2014), while 20E-treated *B. dorsalis* larvae had suppressed transcript levels of *Akt* (Xu K. K. et al., 2015). In the fat body of *B. mori* larvae, application of insulin and methoprene increased *Akt* expression at the active growth period, while 20E decreased *Akt* expression in starved larvae during the terminal growth period (Thounaojam and Keshan, 2017). In *Helicoverpa armigera*, the expression of *phosphoinositide-dependent kinase-1* (*PDK1*, another insulin signaling pathway component) was induced by insulin, but repressed by 20E (Pan et al., 2018). In the present study, the transcript levels of *LsAkt* were upregulated substantially by injection of methoprene or bovine insulin *in vivo*, whereas expression was downregulated by 20E treatment. These results suggest that crosstalk exists among JH, 20E, and insulin signal transduction, but the details are unclear.

The insulin signaling pathway plays crucial roles in insect reproduction. In *D. melanogaster*, insulin signaling directly regulates oocyte growth. Interfering with the insulin cascade blocked the uptake of yolk protein precursors and disrupted oocyte maturation (Das and Arur, 2017). In *M. vitrata*, knockdown of four insulin signaling components (*InR*, *Akt*, *FOXO*, and *TOR*) suppressed *Vg* and *VgR* expression and blocked ovarian development (Al Baki et al., 2019). In *N. lugens*, silencing of *NlInR1* or four insulin-like peptides (*Nlilp1-4*) significantly reduced female fecundity (Xue et al., 2020). By comparing the phenotypes of *T. castaneum* after knockdown of different insulin-signaling genes, the decrease in egg production after the depletion of *InR*, *IRS*, and *TOR* was more severe than after suppression of *PI3K*, *Akt*, and *PTEN* (Parthasarathy and Palli, 2011). In adult females of *Rhodnius prolixus*, depletion of *InR1*, *IGF*, and *ILP1* disrupted the development of ovarian follicles and reduced the numbers of eggs laid (Leyria et al., 2021). In this study, silencing of *LsAkt* significantly decreased *Vg* expression and vitellogenin amount and resulted in atrophied ovaries with less yolk protein deposition. The fecundity and egg hatchability were significantly reduced after knockdown of *LsAkt* expression. Similar defective phenotypes were observed after RNAi of *Akt* in *C. pallens* (Han et al., 2020). Depletion of *Akt* may have inhibited Vg synthesis and ovarian growth, thereby reducing the fecundity of *L. serricorne.*

Insulin signaling principal function in JH metabolism, and its roles have been elucidated in many insects. In *D. melanogaster*, mutations in *InR* or *IRS* caused significant reduction in JH titers (Tatar et al., 2001; Tu et al., 2005). In *Blattella germanica*, silencing of *InR* reduced the mRNA levels of JH biosynthetic enzymes and JH synthesis in corpora allata and affected vitellogensis in adult females (Abrisqueta et al., 2014). In *L. decemlineata*, RNAi of *ILP2* substantially suppressed the expression levels of *JHAMT* and *Kr-h1*, and decreased JH titers (Fu et al., 2016). Depletion of *FOXO*, a transcription factor in the insulin signaling pathway in *B. mori*, induced the expression of three JH degradation pathway genes (Zeng et al., 2017). In this study, we found that the expressions of two JH signaling genes (*LsMet* and *LsKr-h1*) and two JH biosynthesis genes (*LsFAmet* and *LsJHAMT*) were significantly decreased in *LsAkt*-depleted beetles, and expressions of two JH degradation genes (*LsJHE* and *LsJHEH*) were dramatically increased. This indicates that *LsAkt* depletion inhibited JH signaling and activated JH degradation. Accordingly, knockdown of *LsAkt* led to a significant decrease in JH titers. Since JH is a vital hormone promoting Vg uptake in the ovaries, it is possible that decreasing *LsAkt* expression could affect ovarian development.

Carbohydrate metabolism is critical for supplying the energy needed for insect development and reproduction (Hou et al., 2015; Mattila and Hietakangas, 2017). Insulin signaling is functionally related to carbohydrate metabolism. In *D. melanogaster*, ablation of *ILPs* increased carbohydrate levels in the hemolymph, increased lipid storage in the fat body, retarded growth, reduced fecundity, and increased resistance to stress (Rulifson et al., 2002). In *L. decemlineata*, RNAi of *IRS* or *PI3K92E* inhibited the expression of four trehalose metabolic genes (*LdTPS*, *LdTRE1a*, *LdTRE1B*, and *LdTRE2*) and a glycogen synthase gene (*LdGS*), caused a decrease of glycogen accumulation and an increase of glucose and trehalose concentrations, and decreased food consumption (Deng et al., 2018). In *N. lugens*, suppression of *Nlipl1*, *Nlipl2*, *Nlipl3* or *NlInR1* disrupted carbohydrate metabolism and nymph development. However, knockdown of *Nlipl1-3* resulted in increased contents of glucose, trehalose, and glycogen, which contrasts with the effect derived from *NlInR1* knockdown (Xu H. J. et al., 2015). In this study, silencing of *LsAkt* inhibited the expression of *TPS*, increased the transcription level of *TRE*, and caused a dramatic reduction in trehalose content. Trehalose is the energy fuel for Vg formation and oocyte maturation (Lu et al., 2019). This indicates that repression of *Akt* may affect trehalose metabolism during the reproductive process of *L. serricorne*. We also found that the glycogen content was significantly reduced in the *LsAkt* RNAi beetles and the expression level of *LsGlys* was considerably decreased. In *Culex pipiens*, RNAi depletion of *Glys* reduced glycogen and lipid levels and increased the mortality of the diapause females (Olademehin et al., 2020). Studies in *D. melanogaster* revealed that glycogen accumulation is involved in development competence of the oocyte (Sieber et al., 2016). *LsAkt* knockdown severely reduced the expression of a glucose transporter gene (*GLUT*), decreased the expression of a pentose phosphate pathway (PPP) gene (*G6PDH*), and inhibited glucose uptake. *G6PDH* mediates the rate-limiting and committed step of PPP, which provides energy for insect growth and reproduction (Smolinski et al., 2019). Silencing *LsAkt* increased the transcription activation of two glycolytic pathway genes (*LsHK* and *LsPFK*) and lowered the glucose content. These results were not consistent with previous studies. In *B. mori*, downregulated expression of *HK* and *PFK* genes induced by the suppressed *estrogen-related receptor* gene increased glucose levels and influenced embryonic development (Long et al., 2020). In *T. castaneum* and *N. lugens*, silencing of the hexokinase gene increased the glucose amounts and reduced fecundity (Fraga et al., 2013; Ge et al., 2019). The underlying mechanism by which insulin signaling regulates carbohydrate metabolism appears to be complicated and in need of further investigation.

## Conclusion

We obtained a serine/threonine kinase Akt gene *(LsAkt*) from *L. serricorne*. The expression of *LsAkt* was stimulated by methoprene or insulin, while suppressed by 20E exposure. RNAi screening demonstrated that *LsAkt* plays a pivotal role in the ovarian development and fecundity of *L. serricorne*. Knockdown of *LsAkt* inhibited Vg synthesis, disturbed JH production, and disrupted carbohydrate metabolism, resulting in reproductive defects. These results provide fundamental evidence for clarifying regulatory mechanisms of *Akt* in *L. serricorne* reproduction.

## Data Availability Statement

The datasets presented in this study can be found in online repositories. The names of the repository/repositories and accession number(s) can be found below: GenBank, MZ695806.

## Author Contributions

K-KX and HY conceived and designed the experiments and wrote the manuscript. YY, S-YY, and P-LX performed the experiments. YY and K-KX analyzed the data. W-JY, HY, and CL revised the manuscript. All authors gave final approval for the publication.

## Conflict of Interest

P-LX was employed by company Enshi Tobacco Company of Hubei Province. The remaining authors declare that the research was conducted in the absence of any commercial or financial relationships that could be construed as a potential conflict of interest.

## Publisher’s Note

All claims expressed in this article are solely those of the authors and do not necessarily represent those of their affiliated organizations, or those of the publisher, the editors and the reviewers. Any product that may be evaluated in this article, or claim that may be made by its manufacturer, is not guaranteed or endorsed by the publisher.

## Footnotes

1
https://blast.ncbi.nlm.nih.gov/Blast.cgi

2
https://www.ncbi.nlm.nih.gov/orffinder

3
http://www.expasy.ch

4
http://smart.embl-heidelberg.de

## Supplementary Material

Supplementary Figure 1 Nucleotide and deduced amino acid sequences of LsAkt cDNA. The start codon is indicated in bold and the stop codon in bold with an asterisk. The predicted phosphorylation sites, including serine (S), threonine (T), and tyrosine (Y), are marked in red, yellow, and green, respectively. The low complexity region is shaded and PH domain is underlined. The S_TKc domain is boxed and S_TK_X domain is underlined with wavy lines.
