# Supplementary material for: Disruption of the Serine/Threonine Kinase Akt Gene Affects Ovarian Development and Fecundity in the Cigarette Beetle, Lasioderma serricorne
Source: Front Physiol. 2021 Oct 7;12:765819. doi: 10.3389/fphys.2021.765819 (PMC8529032; doi:10.3389/fphys.2021.765819)
Supplement: Supplementary file 1 [file Table_1.DOC]

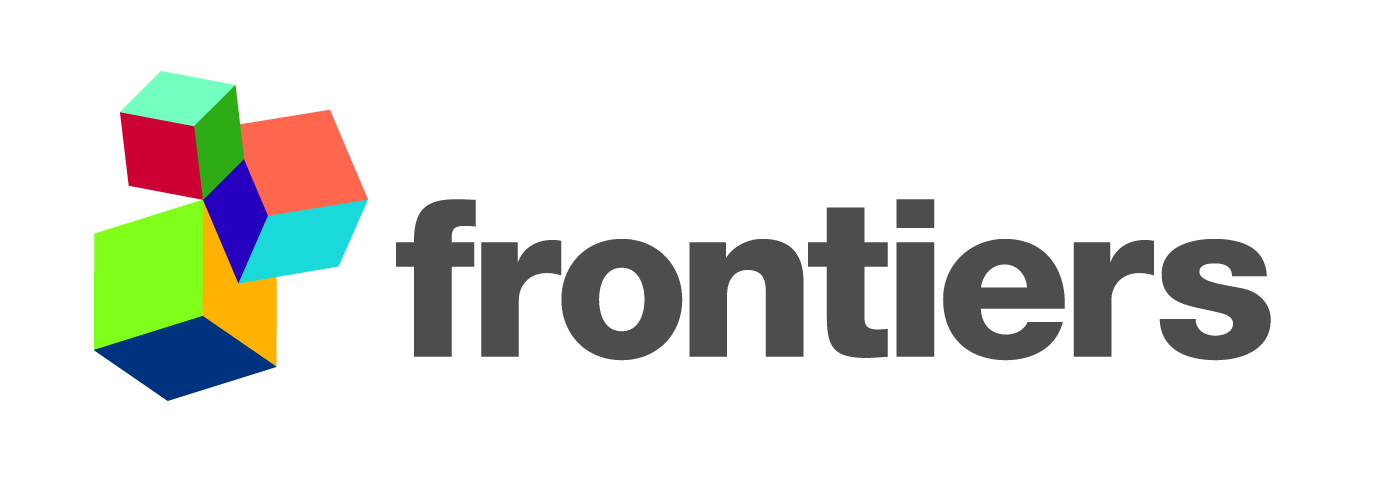
**Table S1. Primer sequences used for cloning, qPCR and dsRNA synthesis**

| **Experiments** | | **Primer Names and Sequence (5′ to 3′)** | | | **size (bp)** |
| --- | --- | --- | --- | --- | --- |
| ORF confirmation | | *LsAkt*-F: AATGCGTTCACTAAAACCGAC | | | 1621 |
|  |  | *LsAkt*-R: ACGTCATGTCAGTCACTGCATG | | |  |
| qPCR analysis | | *LsAkt*-F: GACATGGCATCGAGTGGTCA | | | 206 |
|  |  | *LsAkt*-R: TTAATCGCATATAACCGGCT | | |  |
|  |  | *Ls18S-*F: GTTGATCACGTCGCAAGCTA | | | 208 |
|  |  | *Ls18S*-R: AGGTTTCCCTCTGGCTTGTT | | |  |
|  |  | *LsEF1α*-F: GCATCTCCACGGATTTCACT | | | 213 |
|  |  | *LsEF1α*-R: AAGGCAAGACGCTTATCGAA | | |  |
| dsRNA synthesis | | *LsAkt*-F: taatacgactcactatagggAACCAATGATCGGCTTTGTTTC | | | 474 |
|  |  | *LsAkt*-R: taatacgactcactatagggCTCGCTTCCATGCTAATATTCT | | |  |
|  |  | *GFP*-F: taatacgactcactatagggCAGTTCTTGTTGAATTAGATG | | | 371 |
|  |  | *GFP*-R: taatacgactcactatagggAATGTTACCATCTTCTTTAA | | |  |
| **Gene symbol** | **Gene Name** | | **Forward Primer（5’-3’）** | **Reverse Primer (5’-3’)** | **Size(bp)** |
| **Vitellogenin synthesis and juvenile hormone-related genes for qPCR analysis** | | | | | |
| *LsVg* | *Vitellogenin* | | CGAACCGTCTCTTGCAGTTT | ATGGAAACCGTGAGCAACAG | 214 |
| *LsVgR* | *Vitellogenin receptor* | | CGAGCACTTTCCGTTGTTGA | CTCGCAATCTTTACTGGGCC | 158 |
| *LsIPPI* | *Iisopentenyl diphosphate delta isomera* | | GACATACCGGGTGAAGAGGA | TTCGAGTTCGGTTTCAGCTT | 227 |
| *LsHMGR* | *3-hydroxy-3-methylglutaryl-coenzyme A reductase* | | CCGTTAGTCAGACAGCAGCA | GTTGTCTTGGTCGGGACTGT | 213 |
| *LsAHCy* | *Adenosylhomocysteinase* | | TTTGGGAGCAAATCTTCGTT | TTGAACATTGCGGGGTATTT | 237 |
| *LsFPPS* | *Farnesyl pyrophosphate synthase* | | AGCATCCATCCAATCACCAT | ATGGGAGCCTTCCAACCTAA | 218 |
| *LsJHAMT* | *Juvenile hormone acid O-methyltransferase* | | TCTTGTTGAAAATCGGCCGC | AATCGGATTCCTCGCCAAAA | 169 |
| *LsFAmet* | *Farnesoic acid O-methyltransferase* | | AAGCAGGGAGAGACGCTGTA | TGGTGTGCCATTTTAAACGA | 155 |
| *LsKr-h1* | *Kruppel homolog 1* | | TTTGCGGGAAGTCTTTTGGG | TAACGGAAAGGGAAGGGTG | 249 |
| *LsMet* | *Methoprene-tolerant* | | GAAAGTATGCCGTCCTGAGC | CATGAAATTGAAAGCGGACA | 172 |
| *LsJHE* | *Juvenile hormone esterase* | | CGGTTACAATGATCGCGAGG | TCCATCGAAGGTTCAGTGTC | 177 |
| *LsJHEH* | *Juvenile hormone epoxide hydrolase* | | AGGCGGATTGAAGGGATATGT | TTTCAGGTTGCGAGATGTGC | 151 |
| **Glucose uptake and metabolic genes for qPCR analysis** | | | | | |
| *LsGLUT* | Glucose transporter | | AAGTTTGGCAGGAAAGGTGG | ACGATGTGTTTAAGCCGCAA | 151 |
| *LsG6PDH* | Glucose-6-Phosphate dehydrogenase | | AATTTGGGCCCATAACCGTC | TGGAGGATATTTACGCCGCT | 223 |
| *LsHK* | Hexokinase | | TACGCAGCTTTCCGAGAAGA | CTTTACTGGCATCGACTCGC | 169 |
| *LsPFK* | Phosphofructokinase | | CTGTTCTTGGCCACGTTCAA | AGTTCCCATTGTTTGTCGGC | 226 |
| *LsPK* | Pyruvate kinase | | TACGCAATGTCGGCTCTTTG | CGCCATCACCAATCTAGTAC | 186 |
| *LsGSK-3β* | Glycogen synthase kinase-3 beta | | GCTTCGTGTAATGCCGGAAT | GAAACGACGTTGATGGCCAT | 156 |
| *LsGlyS* | Glycogen synthase | | TGGATTCAAAGGGCTGGCTA | TTAGCTGCCTCCATTGGTGA | 219 |
| *LsGlyP* | Glycogen phosphorylase | | GCGACTGTTAGAGGCTTTGG | CGACAGATGCGCCATGTTAA | 202 |
| **Trehalose metabolic genes for qPCR analysis** | | | | | |
| *LsTPP* | Trehalose-P phosphatase | | TATTCCGTCTCTTCCCGTGG | TTCAGCAAGTTCCACGAAGC | 230 |
| *LsTPS* | Trehalose-P synthase | | CCTCATCCAGATCTCGCCAT | CGGCATTGGATGGACGAATT | 210 |
| *LsTRE* | Trehalase | | AGAGCCTTCGACCAGATACG | GATAAAACACGGTGGAGCCC | 160 |
