# Supplementary material for: Disruption of the Serine/Threonine Kinase Akt Gene Affects Ovarian Development and Fecundity in the Cigarette Beetle, Lasioderma serricorne
Source: Front Physiol. 2021 Oct 7;12:765819. doi: 10.3389/fphys.2021.765819 (PMC8529032; doi:10.3389/fphys.2021.765819)
Supplement: Supplementary file 2 [file Table_2.DOC]

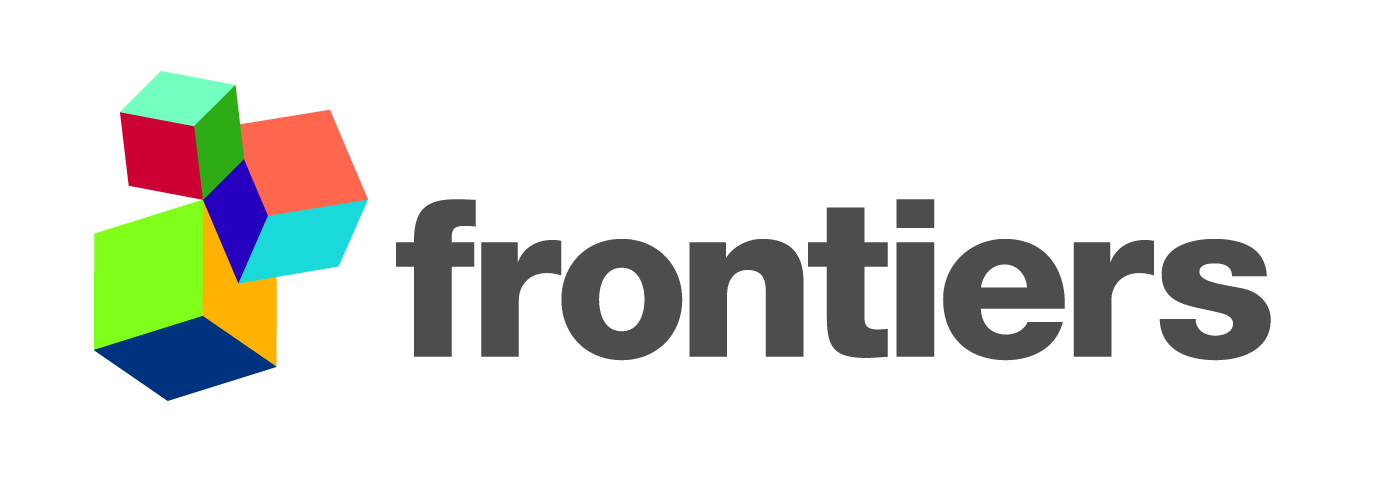
**Table S2. Sequences and relevant information used for phylogenetic and conserved domains analysis of serine/threonine-protein kinases**

| **Species** | **Genbank accession number** | **Length of amino acid sequence** | **Order** |
| --- | --- | --- | --- |
| *Anoplophora glabripennis* | XP_018571651.1 | 513 | Coleoptera |
| *Leptinotarsa decemlineata* | XP_023017656.1 | 517 | Coleoptera |
| *Photinus pyralis* | XP_031339729.1 | 518 | Coleoptera |
| *Agrilus planipennis* | XP_018326395.1 | 522 | Coleoptera |
| *Tribolium castaneum* | XP_008191524.1 | 510 | Coleoptera |
| *Nicrophorus vespilloides* | XP_017772201.1 | 516 | Coleoptera |
| *Sitophilus oryzae* | XP_030750712.1 | 513 | Coleoptera |
| *Glossina fuscipes* | XP_037896199.1 | 524 | Diptera |
| *Aedes aegypti* | AAP37655.1 | 528 | Diptera |
| *Musca domestica* | XP_005186507.1 | 522 | Diptera |
| *Lucilia cuprina* | XP_023308538.1 | 524 | Diptera |
| *Stomoxys calcitrans* | XP_013099148.1 | 522 | Diptera |
| *Culex quinquefasciatus* | XP_001849257.1 | 544 | Diptera |
| *Melanaphis sacchari* | XP_025205153.1 | 521 | Hemiptera |
| *Myzus persicae* | XP_022166962.1 | 522 | Hemiptera |
| *Diuraphis noxia* | XP_015371565.1 | 522 | Hemiptera |
| *Acyrthosiphon pisum* | XP_001951736.1 | 522 | Hemiptera |
| *Bombus impatiens* | XP_003487799.1 | 544 | Hymenoptera |
| *Bombus terrestris* | XP_003399145.1 | 544 | Hymenoptera |
| *Copidosoma floridanum* | XP_023246634.1 | 510 | Hymenoptera |
| *Osmia lignaria* | XP_034193215.1 | 539 | Hymenoptera |
| *Bactrocera latifrons* | XP_018788269.1 | 536 | Diptera |
| *Drosophila melanogaster* | NP_001287353.1 | 530 | Diptera |
| *Rhopalosiphum maidis* | XP_026809886.1 | 522 | Hemiptera |
