# Supplementary figures and images for: Disruption of the Serine/Threonine Kinase Akt Gene Affects Ovarian Development and Fecundity in the Cigarette Beetle, Lasioderma serricorne
Source: Front Physiol. 2021 Oct 7;12:765819. doi: 10.3389/fphys.2021.765819 (PMC8529032; doi:10.3389/fphys.2021.765819)

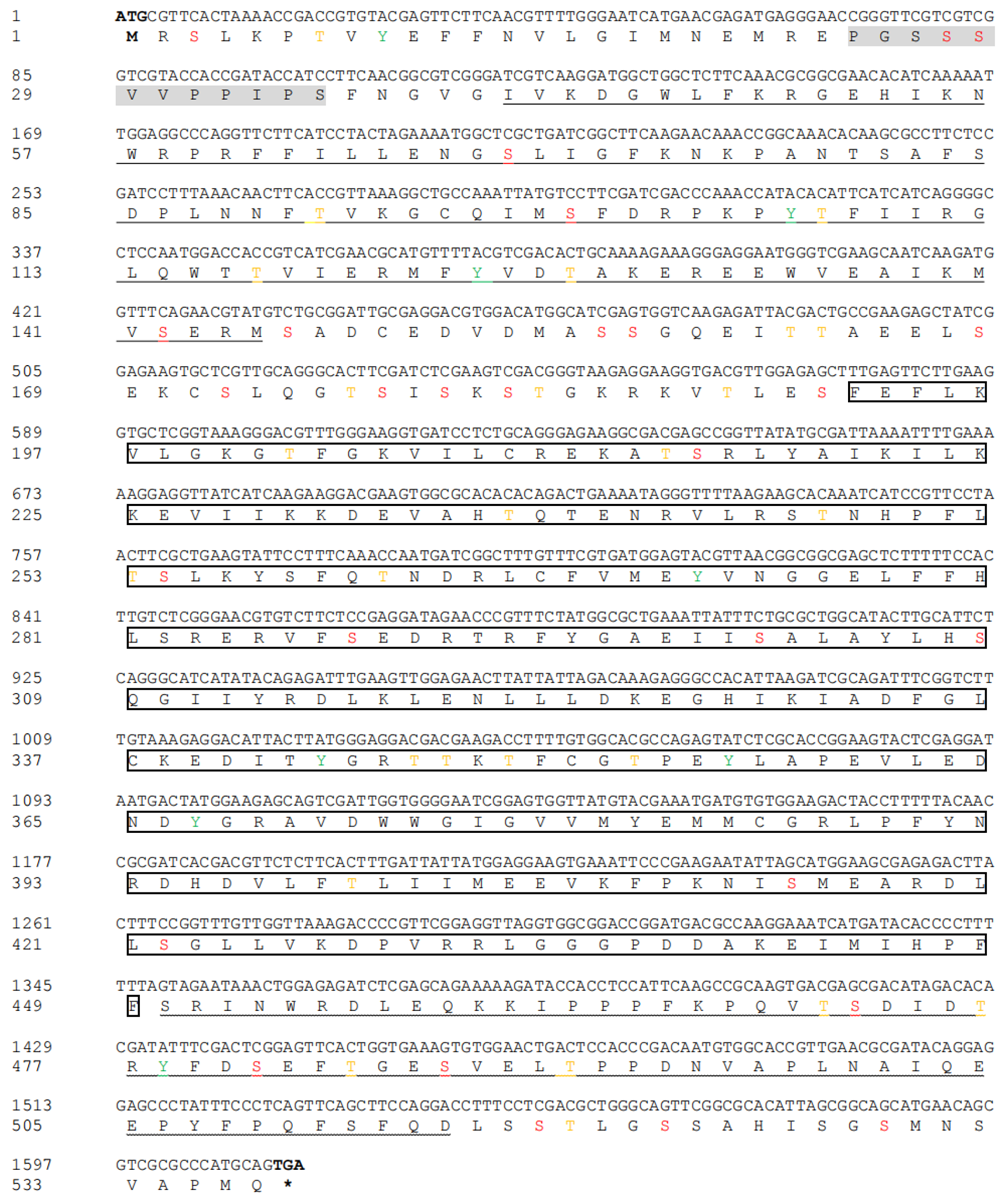

Supplement: Supplementary Figure 1 — Nucleotide and deduced amino acid sequences of LsAkt cDNA. The start codon is indicated in bold and the stop codon in bold with an asterisk. The predicted phosphorylation sites, including serine (S), threonine (T), and tyrosine (Y), are marked in red, yellow, and green, respectively. The low complexity region is shaded and PH domain is underlined. The S_TKc domain is boxed and S_TK_X domain is underlined with wavy lines. [file Image_1.TIF]
